# Fingers and Brains

**Published:** 1887-11-26

**Authors:** 


### The Hospital

A Weekly Institutional Journal of

**Science, Medicine, and Philanthropy.**

*For Hospitals, Asylums, Medical Practitioners, Students, Nurses, Families, Parishes, Congregations, and the Charitable Public.*

Vol. III. No. 61.] [November 26, 1887.

### Fingers and Brains.

It seems a pity, in view of the recent "progress of humanity," that Nature fashioned human beings with such comparatively needless appendages as hands and feet, legs and arms. If she could have foreseen, in the remote ages when we consisted of merely undifferentiated protoplasm, what very clever fellows we should all ultimately become, she would, no doubt, have put her entire energies into the development of a brain pure and simple, and then we should all have been happy. But Nature is, and always has been, proverbially blind, and so left our progenitors to take their chance in the environments in which they found themselves. They, without due consideration of the wonderful possibilities of ages upon ages of time, originated bones and muscles, hearts and lungs, and, above all, stomachs. Now a stomach knows very well how to speak in the various tones of the imperative mood—it "commands, exhorts, or entreats"—but especially "commands." Unhappily, its commands are unheeded except by its possessor; but by him they are felt to be so urgent that nothing short of prompt obedience can even be thought of. A man must eat; and in order to eat he must have the wherewithal. In civilised societies, "ready money" is the grand "open sesame" which clears the way to the cupboard; and he who has that may fold his arms and smoke his pipe in peace. But in the absence of this all-powerful persuader, it behoves the owner of a stomach to bethink himself of ways and means.

There has never been known any period in the world's history when food could be procured by merely wishing for it. "Work and eat"; "Be idle and starve." These are the two evangels which are common to every faith. The happy millennium of reward without labour has not even yet dawned. Science and culture, school boards and churches, Comte and Cardinal Newman are alike powerless to bring about a golden age in which purely intellectual pursuits shall enable us to get rid of quarter day and dispense with the butcher's bill. Work—hard, physical work—is the normal condition of man, be he savage or be he civilised. He must either hunt or fish, or dig up roots and gather berries, if he be savage. If he be civilised, he must plough the ground, sow seed, reap and thresh and grind the corn, or he can by no possibility have bread. So, too, he must make bricks, or quarry stones, or at least cut down and saw and plane wood, if he would have a roof over his head to protect him from the storm. In like manner, clothing of the most rudimentary kind demands the tanning of skins, the shearing of sheep, spinning, weaving, tailoring, and an almost infinite variety of other occupations. The merest elements of logic are sufficient to make it plain to us that man, speaking broadly, is destined to be a working animal, and that his natural and rudimentary tools are his hands. A few individuals, by the energy of their ancestors, may be in a position to let their arms and hands atrophy and degenerate for want of use. But for mankind, as mankind, "work, work, work," is still the unceasing cry.

There is, then, no likelihood of human beings ever developing to such an extent as to consist wholly of pure brain; nor does it seem more probable that the brain will ever become so modified by its environment as to do the work of the hands. So far, at least, as this and one or two succeeding generations are concerned, we may take it for granted that food, and clothing, and house-building, and road-making, and the other thousand-and-one circumstances which go to make civilised life what it is—all will have to be done by hands, and by hands alone. Machinery may help, but it must be guided by the hands.

From a scientific and historical standpoint, the case is as clear as logic can make it, What is the practice in our own country? We pride ourselves mightily upon being a practical people. No mere ideas are ever permitted to run away with us. We do not make abstract constitutions, and compel ourselves to live under them. Our Constitution and laws are the growth of practical necessity. We are a practical people—so we say and think. The thing is not true. We are neither so sensible nor so practical nor so capable as we think ourselves, by a very great deal. Just now we are decidedly playing the fool. We have got an Education Act, and its administrators and their subordinates are rapidly running away with it. Not only so, but we have given up entirely any attempt to provide for the industrial training of the generation that is springing up around us. Apprenticeship is dying out on all sides; and what is taking its place? Nobody knows; nobody cares. Every trade and every individual in it is apparently to be allowed to do exactly what is pleasing in his own eyes. But let every thinking person ask himself this question: Can an individual man hope to bring his life-work to a successful issue without definite purpose and without disciplined work? The man who has no purpose in life, and who practises no discipline, is surely on a downward sliding scale, and is bound to end in failure and disaster. Any man of the least sense and experience knows that. But if that be true of one man, is it not equally true of thirty millions? and if the failure of one man be disastrous, what will the failure of thirty millions be?

The truth is, we want more departmental statesmen: men who have the intelligence and imagination to see the possibilities of such departments as industry and education; and the self-denying patriotism to give themselves up to the constructive and continuous work demanded by such departments; men who have the sense and the manhood to rid themselves of the trammels of party, and to devote themselves to one great end. If a man like the late Professor Fawcett could accomplish in a brief period such great results at a comparatively minor department like the Post Office, what might not an equally honest and capable administrator do in the infinitely larger departments of agriculture, manufactures, and education?

We are all education mad just now. But what is education? Many people, especially schoolmasters, are so stupid that they can see education in the multiplication table, but none in the weeding of an onion bed. He who can show that the angles at the base of an isoceles triangle being equal, the angles at the other side of the base, when the equal sides are produced, shall be equal too, is considered to be on his way to scholarship; whilst he who knows that wheat does best in a heavy soil, and that carrots grow biggest in loam, is held to be uneducated. What arrant nonsense! Who cares whether the angles at the other side of the base of an isoceles triangle be equal or not? But we all care when the bread-loaf is not sufficient to go round the table, and there are no carrots for the soup. It is enough to make a practical man despair to see the schoolmaster with spectacles on nose magnifying his office and teaching his pupils to despise the practical arts of life. If there were no isoceles triangles to be reasoned about, the world would be neither poorer nor less wise; but if there were no cornfields and no bread-loaves, what would become of the schoolmaster and his spectacles?

Do we then despise education? Surely no sane man will ask such a question! But we repeat, What is education? Is it not first of all that which teaches a youth to do his duty to God and his fellow-men; and is it not next that which teaches him to know and do perfectly the particular work by which he has to live? These things *must* be taught; other things *may* be taught if time and opportunity offer. The present system of popular education is robbing the fields and the workshops of those who in fields and workshops would be healthy and prosperous themselves, and would constantly add to the strength and prosperity of the State. At the same time it is filling the country with half-taught clerks and schoolmasters who know nothing of real life and despise honest and manly occupations. These poor simpletons imagine they shall be gentlemen if they wear tall hats and sit at a clerk's desk, although by so doing they cannot earn enough money to satisfy their stomachs with a decent meal. If the middle and upper classes would regard the honest and capable workman with that respect which character and capacity demand, we might hope for a public opinion which would result in the adequate cultivation of our fields, the filling of our workshops, and the increase of a class of workers who in time of peace would maintain the prosperity of the country and in time of war would be its invincible defence.

